# Sensing of Oxygen Partial Pressure in Air with ZnO Nanoparticles

**DOI:** 10.3390/s20020562

**Published:** 2020-01-20

**Authors:** Xin Chang, Shunpu Li, Daping Chu

**Affiliations:** 1 Centre for Photonic Devices and Sensors, University of Cambridge, 9 JJ Thomson Ave, Cambridge CB3 0FA, UK; xc276@cam.ac.uk (X.C.); lishunpu@sztu.edu.cn (S.L.); 2 College of New Materials and New Energies, Shenzhen Technology University, Shenzhen 518118, China

**Keywords:** ZnO nanoparticle, sensor, oxygen partial pressure, thermal annealing, nanofabrication

## Abstract

The demand for sensors in response to oxygen partial pressure in air is increasingly high in recent years and small-size sensors on a micrometer scale and even a nanometer scale are particularly desirable. In this paper, the sensing of oxygen partial pressure in air was realized by a solution-processed ZnO nanoparticle (NP). Thin-film ZnO NP was prepared by spin-coating and a highly sensitive sensor was then fabricated. The oxygen sensing performance was characterized in air and compared with that in nitrogen, which showed an increase in electrical conductance by more than 100 times as a result of decreasing oxygen partial pressure from 10^3^ mBar to 10^−5^ mBar. Moreover, higher sensitivity was achieved by increasing the annealing temperature and the effect of thermal annealing was also investigated. Furthermore, ZnO NP lines with 7 μm in width were successfully patterned with low cost by a mould-guided drying technique from ZnO NP dispersion, which makes ZnO NP extremely promising for miniaturized and integrated sensing applications.

## 1. Introduction

Oxygen gas sensing technology has progressed significantly over the last century and it has been extensively used in various areas such as environmental pollution control [1], physiological process analysis [2,3], health and safety monitoring, etc. [4,5]. Oxygen sensors are also widely used in the automobile industry to control the combustion process by optimizing the air-fuel mixture level [6,7]. In terms of the sensing technologies, different oxygen sensing techniques have been developed including potentiometric equilibrium sensors [1,8,9,10], metal oxide-based oxygen sensors [11,12,13], and optical oxygen sensors [14,15,16]. Greater attention has been given to metal oxide-based semiconducting oxygen sensors due to their advantages including low cost, easy integration, and extensive choices of materials [1,12].

**Figure 1:**
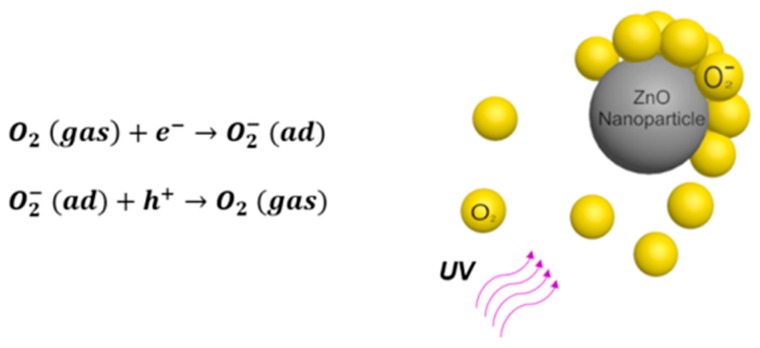
Oxygen adsorption and desorption on ZnO NP.

Zinc oxide nanoparticle (ZnO NP) generally has a particle diameter below 100 nm and it has been attractive to researchers because of its direct wide band gap (3.3 eV), high exciton binding energy (60 meV) at room temperature, and antibacterial properties [17,18]. In addition, ZnO NP is particularly appealing for oxygen sensing due to the large surface-to-volume ratio, high surface reactivity, thermal stability at room temperature, non-toxicity, and eco-friendly nature [17,18,19,20]. It has been widely accepted that ZnO interacts with oxygen in air through the adsorption and desorption processes [21,22,23,24,25,26] (shown in Figure 1). Oxygen molecules adsorb to the surface of ZnO NP by capturing free electrons [27], which forms a highly resistive depletion layer. Oxygen desorption can be achieved by light or heat with sufficient energy such that free electron-hole pairs are generated, releasing adsorbed oxygen molecules.

Various types of ZnO have been attempted by researchers to achieve oxygen sensing. Amorphous ZnO prepared by RF magnetron sputtering (300 °C for 3 h on a glass substrate) was reported to have a limited increase in electrical resistance of less than 10% when the air pressure was dropped from 1 Bar to 1.3 × 10^−6^ Bar [28]. A photo-assisted oxygen sensor was also reported using ZnO thin film (illuminated by white light of 1780 W/m^2^ at 120 °C) with an increase in electrical photoconductivity of 60% (from 1 Bar to 10^−3^ Bar) [29]. ZnO-based nanomaterials were also used for oxygen sensing. A single-crystal ZnO nanowire (NW) produced by chemical vapor deposition was utilized in a field-effect transistor for oxygen sensing and the maximum variation in electrical conductance was 64% under the gate voltage of −1.4 V (from 1 Bar to 1.3 × 10^−5^ Bar) [23]. Enhanced oxygen sensing with a rise in electrical current of 75.4% (from 0.93 Bar to 0.02 Bar) was reported by applying a 0.2% tensile strain to ZnO NW, according to its piezo effect [30]. Pinecone-shaped ZnO was also recorded, which provided a 4% raise in electrical current (1 Bar to 10^−6^ Bar) [31].

In this paper, a solution-processed, low-cost, and highly sensitive oxygen partial pressure sensor based on ZnO NP thin-film was presented. The preparation and characterization of ZnO NP thin films were first discussed in Section 2, which is followed by the fabrication process and sensing performance of ZnO NP-based sensors in Section 3. Subsequently, the sensitivity was shown to be enhanced by thermal annealing of ZnO NP thin films with a discussion on the annealing effect in Section 3.2. Lastly, ZnO NP lines with a micrometer line width were successfully patterned by a mould-guided drying technique. Additionally, the sensing of oxygen partial pressure in air with the patterned ZnO NP lines was demonstrated.

## 2. Methods

**Figure 2:**
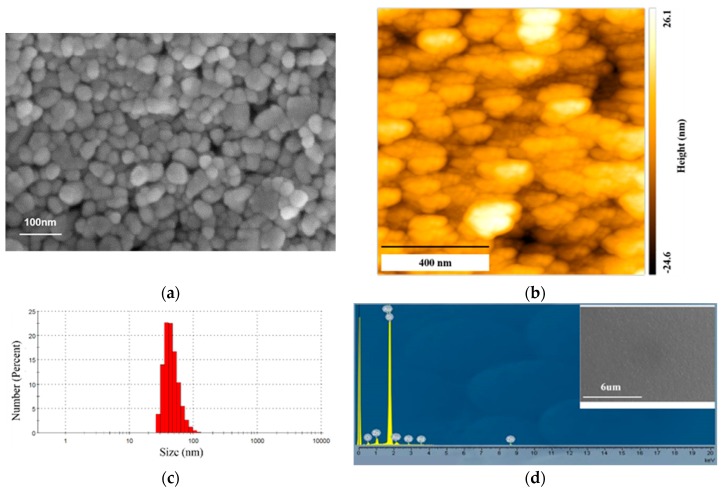
Image of ZnO NPs obtained by (**a**) a scanning electron microscopy and (**b**) an atomic force microscopy, (**c**) a size distribution, and (**d**) EDX spectrum of ZnO NP.

ZnO NP dispersion in ethanol (Sigma Aldrich Co., Gillingham, Dorset, UK, 41 wt.%, 1.25 g/mL) was diluted to 75 mg/mL by ethanol and the ZnO NP thin film was formed on top of SiO_2_ by spin-coating (dispense ZnO NP dispersion on the entire surface of SiO_2_ substrate, which is followed by spin coating with a spin speed of 4000 rpm and acceleration of 500 rpm/s for 30 s). The ZnO NP thin film was inspected by LEO 435 variable-Pressure scanning electron microscopy (VP SEM) (Figure 2a) and atomic force microscopy (Figure 2b). An area of 1 μm × 1 μm was measured by atomic force microscopy (AFM) and the mean surface roughness is 5.8 nm. The particle size distribution was determined by dynamic light scattering (Zetasizer, Malvern, UK), and the average diameter of ZnO NP is 73.2 nm (Figure 2c).

Sample purity and composition was confirmed by energy-dispersive X-ray spectroscopy (EDX) (Figure 2d), which was located in LEO 435 VP SEM. EDX analysis indicates that the ZnO NP film is oxygen-rich (atomic ratio of oxygen and zinc is about 2.05) due to the existence of organic surfactants and oxygen species surrounding ZnO NPs (gold and palladium were deposited on top of the film for a measurement purpose). Two peaks were detected for zinc in the spectrum because electrons from different energy levels (1 keV and 8.6 keV corresponds to the L line and the Kα line, respectively) are recombined with holes at the inner shell.

**Figure 3:**
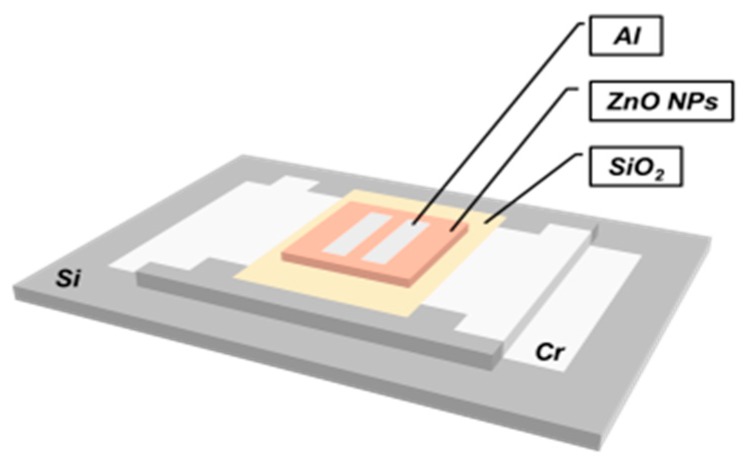
Description of the sensor structure.

An oxygen partial pressure sensor based on a ZnO NP thin film was then fabricated. The schematic illustration of the sensor structure is depicted in Figure 3 below. A silicon substrate was etched by reactive ion etching to form a platform in the center. Then, a thin layer of Cr (100 nm) was deposited by E-beam evaporation. The Cr layer (125 nΩ·m at 20 °C) was patterned in the shape of a ‘bottleneck’ so that a significant amount of heat could be generated locally in a very short period on the etched platform. The etched platform could also keep heat concentrated in the target area. A thin layer of SiO_2_ (100 nm) was further deposited by E-beam evaporation to electrically isolate the power heater (Cr). Then, ZnO NP dispersion was spin-coated in the target area and annealed at 400 °C in air for 3 h. Lastly, Al electrodes (100 nm) were deposited on top by thermal evaporation for the measurement of the electrical conductance of the ZnO NP film (between two electrodes). Aluminum was chosen for avoiding the Schottky junction formed at the metal-oxide interface.

## 3. Results and Discussion

### 3.1. Sensor Performance

**Figure 4:**
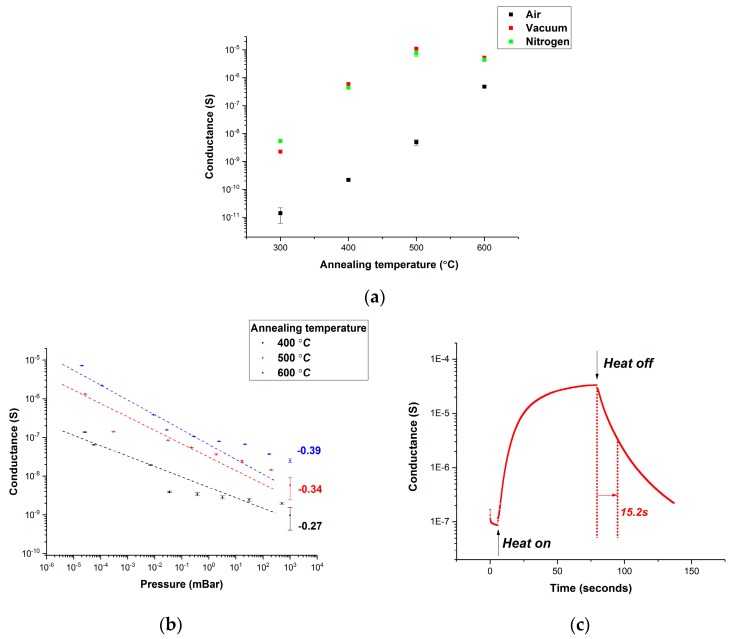
(**a**) Electrical conductance of ZnO NP thin films in different measuring ambient environment. (**b**) Sensing performance for ZnO NP thin films was annealed at 400 °C, 500 °C, and 600 °C for 3 h in air. (**c**) Electrical conductance of ZnO NP thin film when the power heat was on/off.

Electrical conductance of ZnO NP thin film in different measuring ambient environment (air, vacuum, and nitrogen) is shown in Figure 4a. It clearly shows that the conductance is significantly lower in air than in vacuum and in nitrogen. Moreover, the conductance measured in vacuum and in nitrogen is identical (measurement error increases as conductance decreases), which indicates the selectivity of the sensor. The relationship between electrical conductance of ZnO NP thin film and oxygen partial pressure in air is depicted in Figure 4b. It can be observed that ZnO NP thin film has a higher electrical conductance at lower oxygen partial pressure and the conductance increases by more than 100 times when the pressure drops from 10^3^ mBar to 10^–5^ mBar. Figure 4c shows the heat erasing feature of the sensor. The current surges dramatically once the power heat is on because thermal energy frees electrons from oxygen adsorption. Current drops after the heat is off and the off time is about 15.2 s (from 90% to 10%).

The sensitivity can be described by the relationship between oxygen partial pressure ($P_{O_{2}}$) and electrical conductivity (σ) [1,13].

(1) $$\sigma =Aexp^{\left(-\frac{E_{a}}{kT}\right)}P_{O_{2}}^{m}$$

where *E_a_* is trap activation energy and *m* is dependent on the charge carrier type and defects. The absolute value of the power index (*m*) quantitatively describes the sensitivity to oxygen partial pressure. The power index (*m*) is extracted by linear fitting and it is −0.27 for ZnO NP annealed at 400 °C (conductance in air was not used due to the large measurement error). Figure 4b also shows the sensitivity of ZnO NP thin film annealed at 400 °C, 500 °C, and 600 °C in air for 3 h (*m* values are calculated to be −0.27, −0.34, and −0.39, respectively). It clearly shows that the power index (*m*) increases with higher annealing temperature and the best sensitivity to oxygen partial pressure (*m* = −0.39) was realized at 600 °C.

### 3.2. Effect of Thermal Annealing

It has been proved in Section 3.1 that thermal annealing has a positive effect on the sensitivity of ZnO NP thin film to oxygen partial pressure in air. This is due to an increased amount of free charge carriers in ZnO NP after thermal annealing. Higher charge carrier density in ZnO NPs increases the capacity for oxygen adsorption and, thus, enhances the sensitivity to oxygen partial pressure in air.

**Figure 5:**
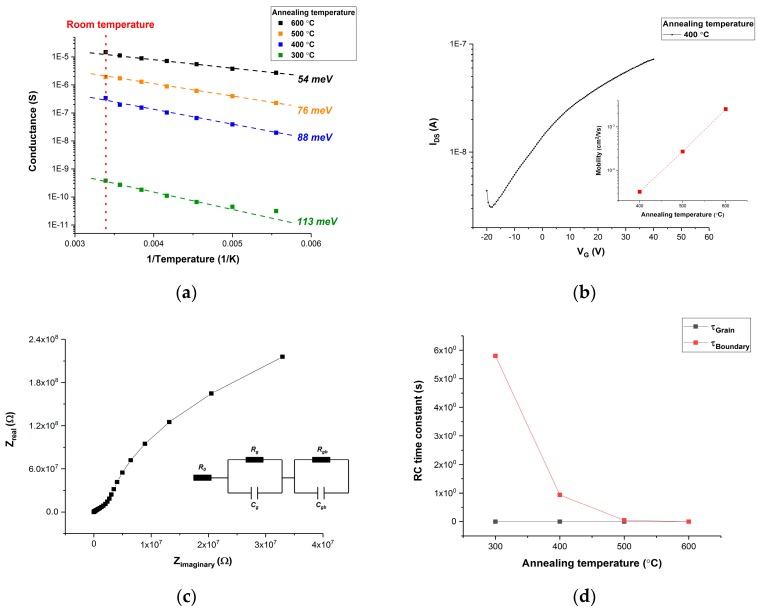
(**a**) Arrhenius plot of ZnO NP thin film annealed at 300 °C, 400 °C, 500 °C, and 600 °C for 3 h in air. (**b**) The transfer function of ZnO NP thin film transistor and field-effect mobility of ZnO NP prepared at different annealing temperatures. (**c**) Nyquist plot and equivalent circuit model of ZnO NP. (**d**) Annealing effect on RC (resistor and capacitor) time constants.

Electrical conductance can be used to indicate the free charge carrier density. ZnO NP thin films were prepared by spin-coating on top of SiO_2_ substrate (with doped silicon on the bottom). Aluminum electrodes (100 nm thick, 80 μm between electrodes) were thermally deposited on top of ZnO NP thin film after thermal annealing at 300 °C, 400 °C, 500 °C, and 600 °C for 3 h in air. The measurement was carried out at room temperature by Agilent 4156 (Yokogawa-Hewlett-Packard Ltd., Tokyo, Japan) and the result is shown in Figure 5a (red dotted line in a vertical direction) below. It is clear that higher annealing temperature leads to larger electrical conductance.

Moreover, the density of free charge carriers is also influenced by trap states. Trap states with larger trap activation energy leads to a less free charge carrier density because trapped charge carriers do not contribute to electrical conductance. The trap activation energy (*E_a_*) can be determined by a low-temperature measurement and it was conducted by a Lake Shore cryogenic probe station (with liquid nitrogen). Electrical conductivity (σ) is represented by the equation below.

(2) $$\sigma =\sigma_{0}\left(exp^{\left(\frac{E_{a}}{kT}\right)}\right)$$

where σ_0_ is a constant and the trap activation energy (*E_a_*) can be extracted by the slope of the Arrhenius plot (1/T, Log (σ)). The result is shown in Figure 5a. It is evident that trap activation energy becomes shallower at higher annealing temperature (from 113 meV at 300 °C to 54 meV at 600 °C).

Thermal annealing also removes the impurities in ZnO NP thin film such as organic surfactant. This can be indicated by charge carrier mobility (μ) because impurities have a negative effect on charge carrier mobility due to scattering. The field effect mobility of ZnO NP thin film was measured with the same sample structure. The transfer function of the ZnO NP thin-film transistor is shown in Figure 5b above. The field effect mobility of ZnO NP annealed at 400 °C was calculated to be 3.23 × 10^−5^ cm^2^/Vs. Higher mobility was noticed at a higher annealing temperature, which is shown in the inset of Figure 5b.

This is further confirmed by impedance spectroscopy, which is a useful tool to investigate the charge transport within ZnO NP thin film. Impedance spectroscopy of ZnO NP thin film annealed at different temperatures (from 300 °C to 600 °C) was performed by Autolab PGSTAT302 (bias voltage of 1V and excitation voltage of 10 mV) and the Nyquist plot is shown in Figure 5c. The equivalent circuit model of ZnO NP can be derived, according to the number of semicircles in the Nyquist plot. It is evident that the Nyquist plot consists of two semicircles, which indicates two RC parallel circuits in series (inset of Figure 5c). Two RC parallel circuits correspond to the grain and boundary of ZnO NP [27,32]. Resistance R_0_ stands for electrode and cable resistance. Each RC parallel circuit is associated with an RC time constant, which describes the time for polarized charge carriers to reach equilibrium. RC time constants of two RC parallel circuits are calculated by fitting the Nyquist plot and they are plotted against annealing temperature in Figure 5d. It is illustrated that thermal annealing shortens the RC time constant of the boundary from 5.8 s (300 °C) to 1.9 ms (600 °C). The decrease in the RC time constant indicates that thermal annealing results in a better charge transport at the boundary of ZnO NP. In contrast, thermal annealing has little effect on the grain of ZnO NP. The RC time constant of ZnO NP grain stays at 3.7 μs.

### 3.3. Sensing with the Patterned ZnO NP Lines

The demand for the miniaturized oxygen partial pressure sensor is becoming increasingly high due to the low signal-to-noise ratio, low power consumption, reduced weight, and low cost, especially for integrated applications [1,33,34]. The patterning of ZnO NPs have been attempted by various techniques such as inkjet printing [35], imprinting [36], laser ablation [37], and photolithography [37,38]. However, the patterning of ZnO NPs is still challenged by the mechanical stability, chemical resistivity, and cost, etc. In this case, we used a low-cost line patterning technique for solution-processed ZnO NP. Mould guided drying [39] and the sensitivity of the patterned ZnO NP lines to oxygen partial pressure in air was also measured.

**Figure 6:**
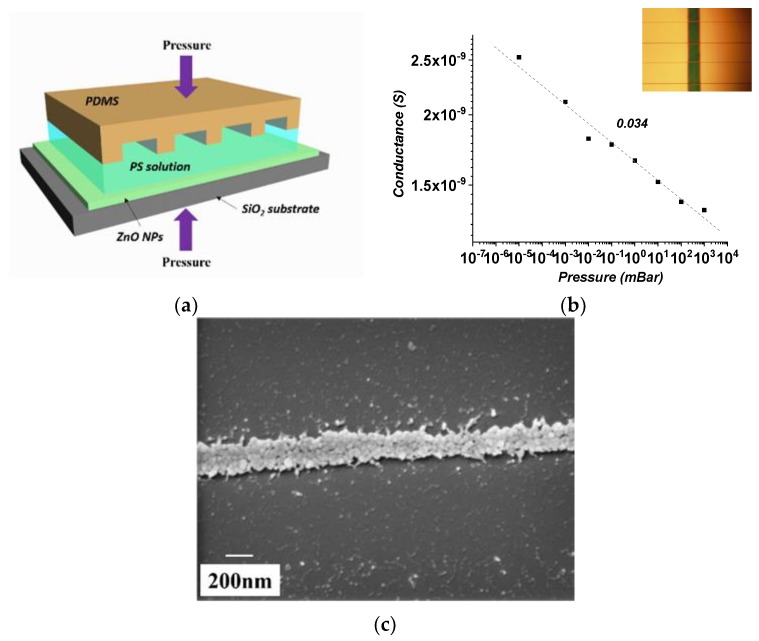
(**a**) Illustration of mould-guided drying technique. (**b**) The sensing of oxygen partial pressure in air with patterned ZnO NP lines. (**c**) ZnO NP lines with less than 200 nm in width.

ZnO NP thin film was first spin-coated on SiO_2_ substrate and then annealed in air at 400 °C for 3 h. A reusable polydimethylsiloxane (PDMS) mould was pre-fabricated by pouring commercial silicone elastomer (Sylgard^®^184, Dow Corning, Barry, UK) onto a photoresist master (made by optical lithography), and then baking at 70 °C for 1 h Then, polystyrene (PS) lines were formed on top of ZnO NP thin film by a PDMS mould, as is shown in Figure 6a. A drop of PS solution in 1,2-dichlorobenzen (DCB, 0.4 mg/mL) was casted on top of the PDMS mould and the mould was brought into contact with the substrate (covered with the ZnO NP thin film) by a customized stamp. PS lines were annealed in air at 100 °C for 1 h. The substrate was then immersed in diluted hydrochloric acid (HCl, 0.5%) for 2 s to remove the ZnO NPs that was not covered by PS lines. Lastly, the sample was treated in oxygen plasma for 5 min to remove the residual PS. The width of the patterned ZnO NP lines was measured to be about 6.5 μm. Aluminum electrodes were deposited as electrodes (inset of Figure 6b) and the sensing performance of oxygen partial pressure in air is shown in Figure 6b. The power index m is extracted to be −0.034, which is less than ZnO NP thin film annealed under the same temperature (*m* = −0.27). However, the conductance (in log) and partial pressure has a better linearity compared to thin films. Nanoscale ZnO NP lines were achievable by reducing the concentration of PS solution. Figure 6c shows the ZnO NP lines with the line width of less than 190 nm. Nanopatterning of ZnO NPs makes this technique extremely suitable for miniaturized and integrated systems. In addition, this technique has a good reproducibility, low cost, and great potential for large-scale production.

## 4. Conclusions

Highly sensitive oxygen partial pressure sensor based on ZnO NP thin film was presented in this paper. The sensitivity in ambient air was improved by increasing the annealing temperature due to better electrical conductance after thermal annealing. The selectivity was demonstrated by comparing ambient nitrogen. It was observed that thermal annealing results in larger conductance at the boundary of ZnO NP, higher charge carrier mobility, and shallower trap states. The micropatterning of ZnO NPs was also achieved using a mould-guided drying technique. The oxygen partial pressure sensors based on ZnO NPs lines exhibited a smaller sensitivity and a better linearity, which is particularly important for integrated applications.
